# The Influence of Nano-Fe_3_O_4_ on the Microstructure and Mechanical Properties of Cementitious Composites

**DOI:** 10.1186/s11671-016-1401-1

**Published:** 2016-04-11

**Authors:** Pawel Sikora, Elzbieta Horszczaruk, Krzysztof Cendrowski, Ewa Mijowska

**Affiliations:** Faculty of Civil Engineering and Architecture, West Pomeranian University of Technology, Al. Piastow 50, 71-311 Szczecin, Poland; Institute of Chemical and Environment Engineering, West Pomeranian University of Technology, Pulaskiego St. 10, 70-322 Szczecin, Poland

## Abstract

In the last decade, nanotechnology has been gathering a spectacular amount of attention in the field of building materials. The incorporation of nanosized particles in a small amount to the building materials can influence their properties significantly. And it can contribute to the creation of novel and sustainable structures. In this work, the effect of nano-Fe_3_O_4_ as an admixture (from 1 to 5 wt.% in mass of the cement) on the mechanical and microstructural properties of cementitious composites has been characterised. The study showed that Fe_3_O_4_ nanoparticles acted as a filler which improved the microstructure of a cementitious composite and reduced its total porosity, thus increasing the density of the composite. The presence of nanomagnetite did not affect the main hydration products and the rate of cement hydration. In addition, the samples containing nanomagnetite exhibited compressive strength improvement (up to 20 %). The study showed that 3 wt.% of nano-Fe_3_O_4_ in the cementitious composite was the optimal amount to improve both its mechanical and microstructural properties.

## Background

Concrete is the most widely used material in the world. Its primary ingredient, cement, is also the most costly and environmentally unfriendly component in the concrete mix. The cement industry is one of two primary industrial producers of carbon dioxide (CO_2_), creating up to 5 % of worldwide man-made emissions of this gas. Therefore, additives and admixtures are widely used in order to reduce the quantity of cement used and to obtain concrete of the same quality.

The development of nanotechnology in the last decades has enabled researchers to apply nanosized admixtures which can improve concrete properties more efficiently than conventional products. This improvement is attributed to the unique properties of nanomaterials, such as their high strength, high Young’s modulus, high surface area, electrical conductivity and certain chemical activity. The industrial production and incorporation of nanomaterials into concrete is undoubtedly the future of modern concrete technology. Nanomaterials are not only environmentally friendly, they can also help to create novel, sustainable and advanced concrete structures, resulting in lowering the use of cement and decreasing project costs.

Among the most promising nanomaterials, nanosilica and titanium dioxide are the most popular in concrete applications due to their unique properties [1–3]. In general, the effects of nanomaterials on the performance of cement-based materials are reflected in their enhancement of concrete strength (compressive and flexural), refinement of microstructure (reduction of total porosity), acceleration of calcium silicate hydrate (C-S-H) gel formation and the enhancement of Young’s modulus [1, 4–6]. In addition, the application of certain nanomaterials allows cementitious composites to exhibit self-cleaning and self-sensing properties [6, 7].

Even though certain nanomaterials have been extensively studied, there is still a high amount of available nanomaterials which influence the properties of concrete still need to be revealed. One of the most promising nanomaterials which should be further investigated is nano-Fe_3_O_4_ (nanomagnetite). The studies related to the application of iron oxides (especially Fe_2_O_3_) in cementitious composites have shown that these nanomaterials positively influence the mechanical and microstructural properties by improving compressive and flexural strength and by reducing the total porosity of the composites [8–13]. In addition, application of nano-Fe_2_O_3_ can be very beneficial in improving the self-sensing properties of concretes [7].

Moreover, the researchers confirm that the application of iron oxides in a dispersed phase may also play an important role in the future production of heavyweight concretes [1], which can find potential applications in—inter alia—shielding concretes. Studies related to the influence of Fe_3_O_4_ on the shielding properties of concretes have shown very successful results [14]. However, due to the high surface energy of iron oxides, these particles have a tendency towards agglomeration, which may lead to the microcracking and strength deterioration if high amounts of nanoparticles would be applied [9].

Data related to the influence of nano-Fe_3_O_4_ is very limited and therefore there is still plenty of room to investigate the influence of this nanoparticle in cement-based composites; this being the aim of this study. Researchers report that the application of nano-Fe_3_O_4_ in small amounts (up to 0.3 wt.%) can lead to the enhancement of mechanical properties and refinement of the pore structure [8, 15, 16]. Other reports ascertain that that introduction of 1.5 wt.% of nano-Fe_3_O_4_ improves compressive strength as well as reducing chloride penetration and water absorption. In addition, studies related to the influence of iron oxides on the behaviour of cement pastes at elevated temperature have shown promising results [17].

The aim of the present study is to characterise the effect of nano-Fe_3_O_4_ as admixture on the mechanical and microstructural properties of cementitious composites. Additionally, the influence of nano-Fe_3_O_4_ on the hydration process and the microstructure of cementitious composites will also be determined.

## Methods

### Composition of the Cement Mortars

The Rapid Hardening Portland Cement (RHPC) type I 42.5R was used as received. Its chemical composition is reported in Table 1. Additionally, for the cement mortar preparation, 0/2 mm of the fine aggregate quartz sand and tap water was used. The water to cement (w/c) ratio was fixed at 0.5 and the aggregate to cement (a/c) ratio was fixed at 3 to enable a reasonable composite workability. No additional admixtures and additives were used in order to solely determine the influence of the nanomagnetite presence in the cementitious composite.

**Table 1:** Chemical composition of the portland cement (wt.%)

Material	SiO_2_	Al_2_O_3_	Fe_2_O_3_	CaO	MgO	SO_3_	Na_2_O	K_2_O	Cl^−^	Loss on ignition
CEM I 42.5R	19.5	4.9	2.9	63.3	1.3	2.8	0.1	0.9	0.06	3.5

Before the introduction of the nanomaterials into the cement mortars, the nano-Fe_3_O_4_ particles were sonicated in water for 1 min to obtain a uniform dispersion. The nanomagnetite structures in size of 50–100 nm (purity 97 %) were purchased from Sigma Aldrich (637106) and were used as received.

### Characterisation of the Nanomaterials and the Cement Composites

The identification of the crystallographic phase of the iron oxide nanoparticles and cement composites was performed using an X-Pert Philips PRO X-ray diffractometer and CoKa radiation. The X-ray diffraction (XRD) of the cement composites was collected after 7 and 28 days of curing. High-resolution transmission (HR-TEM) and scanning (SEM) electron microscopic investigations were conducted using a Fei Tecnai G2 F20 STwin coupled with an energy-dispersive X-ray (EDX) spectroscopy and a Hitachi SU 8000, respectively.

The consistency of the fresh mortars was determined by the flow table method according to EN 1015-3 standard. The fresh mortar was poured into oiled moulds to form samples with a size of 40 mm × 40 mm × 60 mm. The samples were then de-moulded after 24 h and cured for 28 days in a standard water bath at a temperature of 20 °C ± 2 °C. After 28 days of curing, the flexural and compressive strengths of the samples were determined. Six mortar bars were tested for flexural strength and 12 for compressive strength, and the average strength values were obtained. The test (mixing method, curing conditions, testing times) was carried out in accordance with EN 196-1 standard.

For the characterisation of the pore structure, mercury intrusion porosimetry (MIP) was applied. The MIP method is a common method used to characterise the pore structure in porous materials due to its simplicity, quickness and wide measuring range of the pore diameter [9]. In order to provide the information about the pore size distribution of cement mortars, MIP test was performed on small-cored samples taken out from the specimens. After 28 days of curing, the samples were transferred to a freeze dryer to stop the hydration and remove moisture of the pores.

The calorimetry test was carried out using a BMR differential microcalorimeter, at 22 ± 2 °C for a maximum of 72 h. In this test, 25 g of cement was mixed with water and the admixture and the samples were then transferred to the cell.

## Results and Discussion

**Fig. 1:**
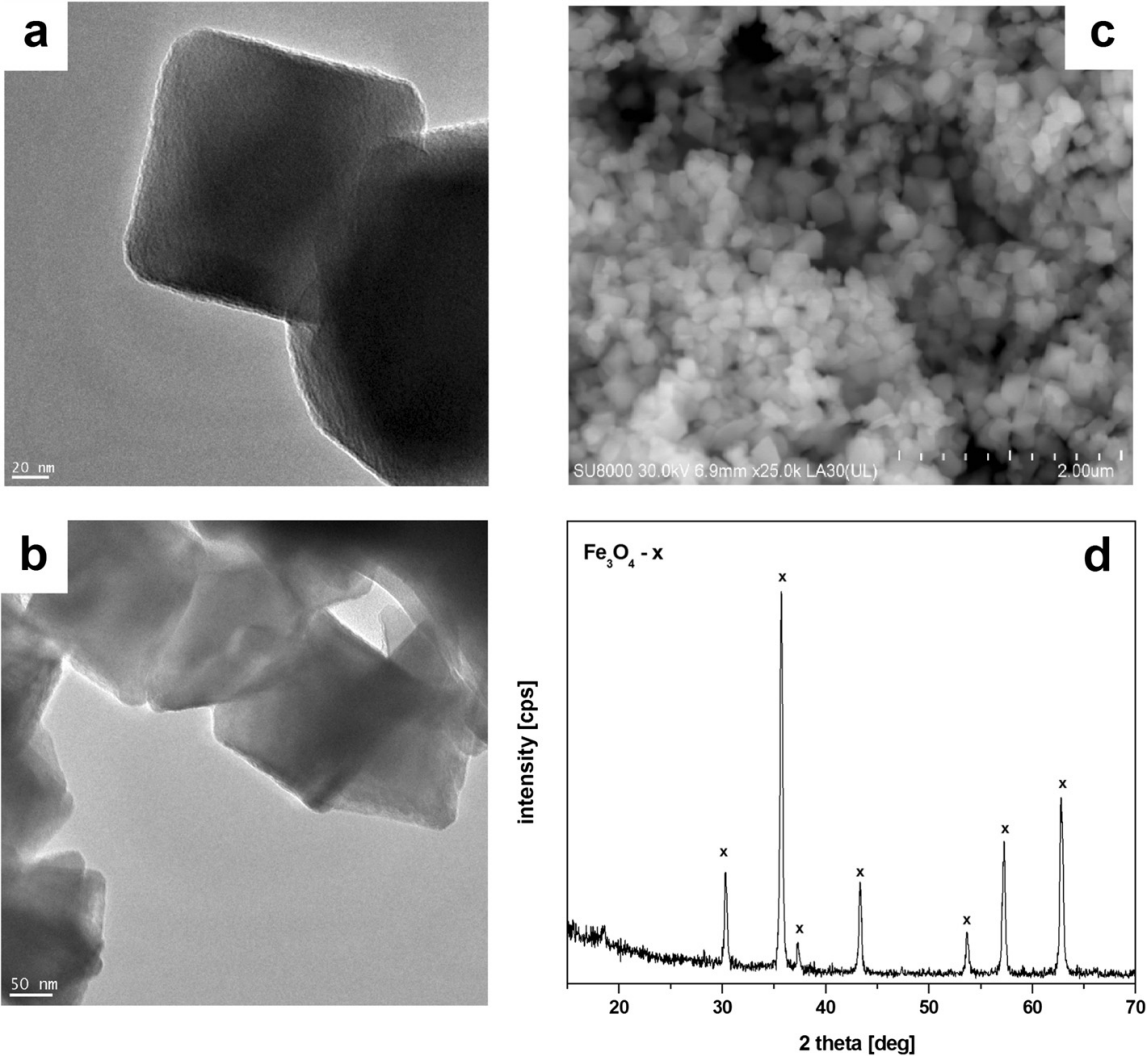
TEM (**a**, **b**), SEM (**c**) micrograph and X-ray diffraction pattern (**d**) of the nano-Fe_3_O_4_

Transmission electron micrographs (TEM) and powder X-ray diffraction (XRD) diagrams of the Fe_3_O_4_ nanoparticles are shown in Fig. 1. The SEM (Fig. 1c) and TEM (Fig. 1a, b) images of the magnetite show that the iron oxide nanoparticles are in a cubic shape with a diameter in the range of 50–100 nm. The particle size distribution has been provided by the producer of nano-Fe_3_O_4_ (Sigma Aldrich—637106) and confirmed by authors with the use of TEM. The magnetite phase of the nanosized iron oxide was confirmed via XRD (Fig. 1d). The diffraction pattern of iron oxide shows seven characteristic diffraction peaks corresponding to the standard card—JCPDS 19-629: 30.28 (30.14); 35.70 (35.52); 37.29 (37.0); 43.06 (43.31); 53.49 (53.7); 56,98 (57.28) and 62.75 (62.53). Difference between the measured and standard peaks is insignificant and proves the magnetite phase.

**Fig. 2:**
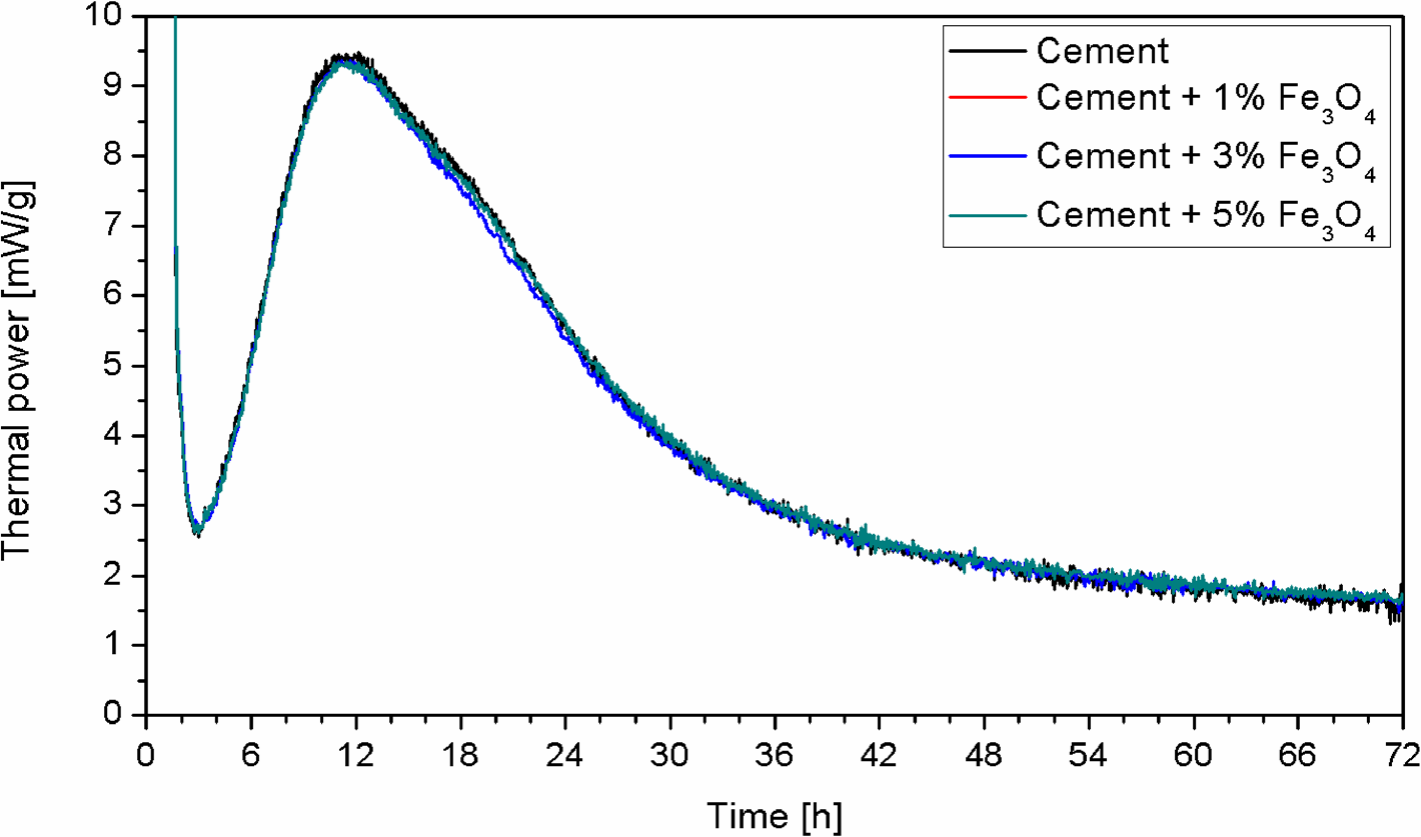
Heat flow calorimetry of the cement with different dosages of nano-Fe_3_O_4_

**Fig. 3:**
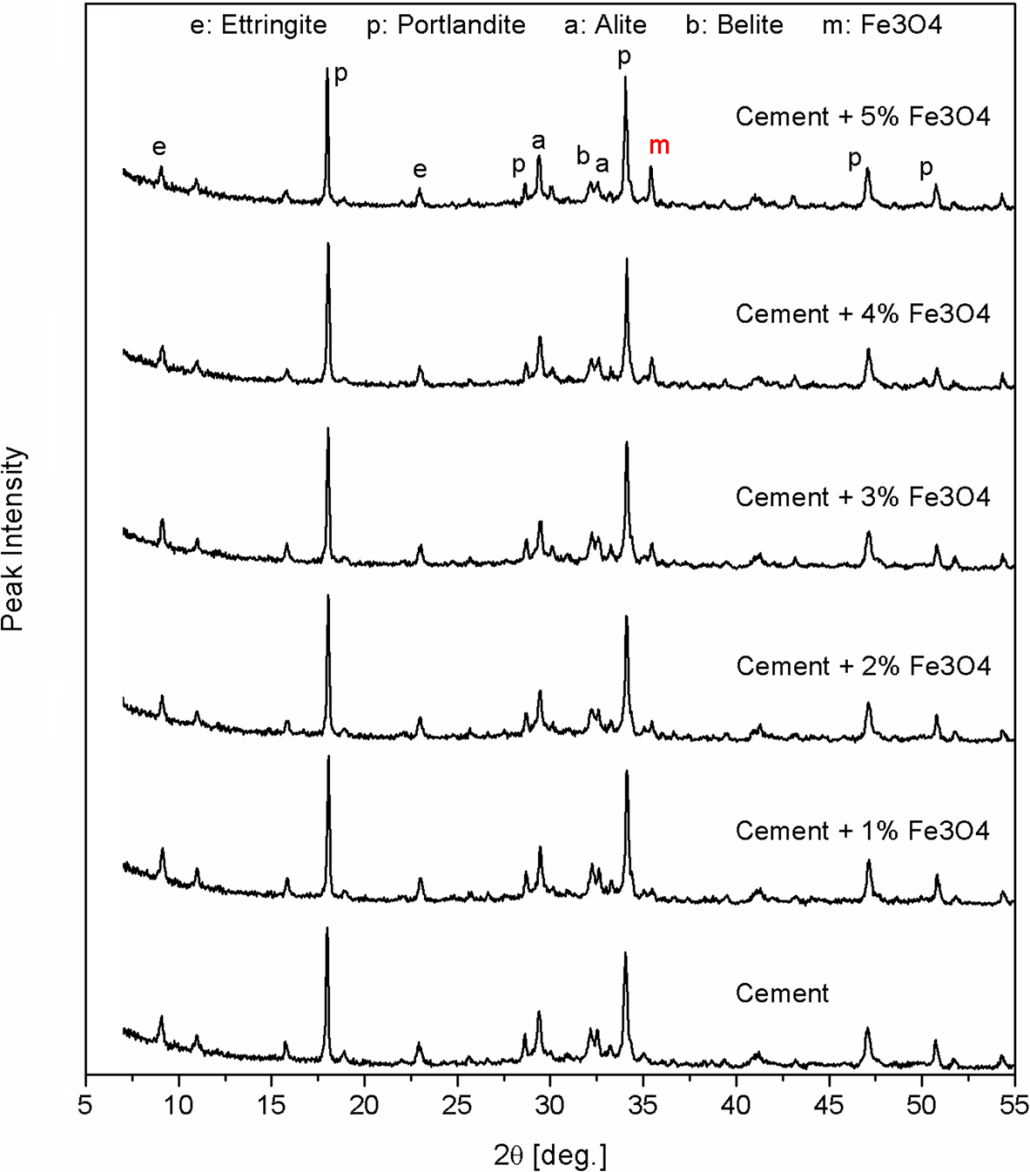
XRD spectra of the cement pastes containing nanomagnetite after 7 days of curing

The normalised heat flow curves of the cement pastes containing 0, 1, 3 and 5 wt.% of nano-Fe_3_O_4_, respectively, are presented in Fig. 2. The conduction calorimetry test indicated that the presence of nano-Fe_3_O_4_ did not affect the heat release during 72 h of hydration. The XRD of the hardened pastes after 7 days is shown in Fig. 3. It can be noticed that the main phases are attributed to calcium silicate hydrates (C-S-H), portlandite (CH) and CaCO_3_. Moreover, with the increased content of nano-Fe_3_O_4_ particles, the peaks related to the presence of magnetite are enhanced. The XRD obtained after 7 days of curing displayed the same hydrate phases as the reference sample. Likewise, after 28 days of curing, no phase changes occurred. Therefore, the addition of nanomagnetite powder did not affect the main hydration products (calcium silicate hydrates and portlandite). These results confirm the observations of Amin et al. [15] and indicate that nano-Fe_3_O_4_ particles have no effect on the rate of cement hydration, as well as on the nature of the formed hydration products.

### Properties of the Fresh and Hardened State—Consistency

**Fig. 4:**
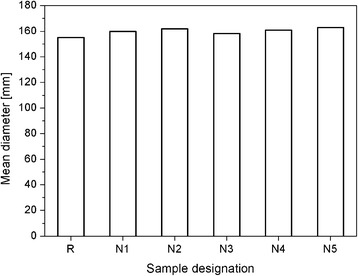
Consistency of the fresh mortars

The consistency of the fresh mortars was determined by the flow table method, and the results are presented in Fig. 4. As can be noticed, the addition of nano-Fe_3_O_4_ did not influence the consistency of the fresh mortars. This can be attributed to the non-porous morphology of Fe_3_O_4_ and its high hydrophobic character in comparison with other nanomaterials applied in cementitious composites [18]. The most popular nanoparticles currently used in concrete technology (such as nanosilica and titanium dioxide) are mostly porous and exhibit a natural hydrophilic nature. Therefore, a high specific surface area and a high water demand (associated with the surface area) cause a reduction of the water that is free for the hydration process, thus leading to limited workability [1]. In the presented results, nanomagnetite (which is characterised by a relatively low surface area and non-porous structure) did not affect the workability of the fresh mortars.

### Flexural Strength and Compressive Strength

**Fig. 5:**
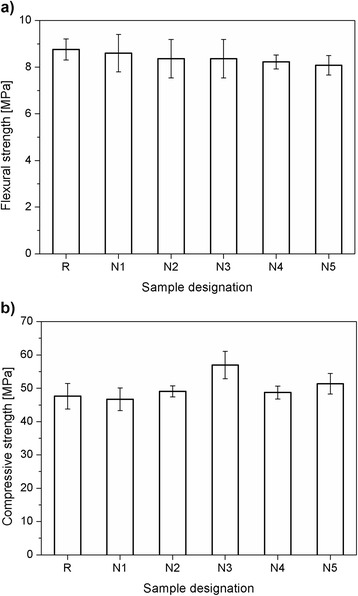
Flexural strength (**a**) and compressive strength (**b**) of the cement mortars after 28 days of curing

The results of the flexural strength and compressive strength tests are depicted in Fig. 5. It can be clearly seen in Fig. 5a that with the increase of nano-Fe_3_O_4_ content, the flexural strength decreases. However, this decrease is not very significant. The samples containing a higher content of the nanomagnetite also had a tendency to exhibit a lower strength due to the nonhomogeneous dispersion of the nanoparticles in the cement paste [12].

The effect of the presence of nano-Fe_3_O_4_ is more enhanced in the case of the compressive strength as a main parameter describing the concrete properties. Figure 5b shows that a small amount of nano-Fe_3_O_4_ does not significantly influence the compressive strength of the cement mortars. However, with the increase of the nanoadditive to 2 or 3 wt.%, a positive effect on the compressive strength is detected. The highest compressive strength was obtained for the samples containing 3 wt.% of nano-Fe_3_O_4_ (N3). However, the increase of the nano-Fe_3_O_4_ to 4 or 5 wt.% does enhance the compressive strength, but a reduction in the strength can be noticed. Therefore, in the case of our study, it seems that the sample containing 3 wt.% of nano-Fe_3_O_4_ (N3) is optimal. These results have been confirmed by the previous findings of Yazdi et al. [11] and Amin et al. [15], which show that there is a certain amount of Fe_2_O_3_ or Fe_3_O_4_ that is beneficial for cementitious composites, and exceeding this amount might result in the lowering of the strength of the cementitious composites.

Two phenomena could be responsible for the positive influence of nano-Fe_3_O_4_ on the compressive strength of the cementitious composites [19]. Firstly, it is known that the fine particle size of the components in the cement paste can significantly affect the hydration kinetics of the cement. Due to their stronger electrostatic attractive forces and a greater specific surface area, a more rapid setting and hardening of the modified cement paste can be obtained [17]. Therefore, fine particles of nanomaterials can accelerate the cement hydration due to their high level of activity. This effect was described by Amin et al., where a small amount (up to 0.3 %) of ultrafine Fe_3_O_4_ nanoparticles was applied to a cementitious composite [15]. When the hydration process started to occur, the hydrate products diffused and enveloped the ultrafine nanoparticles, acting as the kernel. If the amount of the nanoparticles is optimal, the crystallisation will be controlled and the growth of Ca(OH)_2_ crystals will be prevented by the nanoparticles; thus, the improvement of the microstructure of the cement paste will be observed. However, when the amount of nanoparticles exceeds the required limit, Ca(OH)_2_ crystals cannot grow up enough due to the limited space. The decreased ratio of the crystals to the strengthening gel leading to an increase of shrinkage and creep in the cement matrix is also observed. Hence, the pore structure of such a cement paste is relatively loose [9, 19].

Secondly, due to their ultrafine size, the nanoparticles fill the pores (the nanofiller effect), leading to the further compacting of the microstructure [11]. These two main phenomena lead to the improvement of the microstructure by reducing the amount of pores, improving the bond between the aggregate and the cement matrix and increasing the density of the cementitious composite [9].

### Microstructure and Pore Structure

**Fig. 6:**
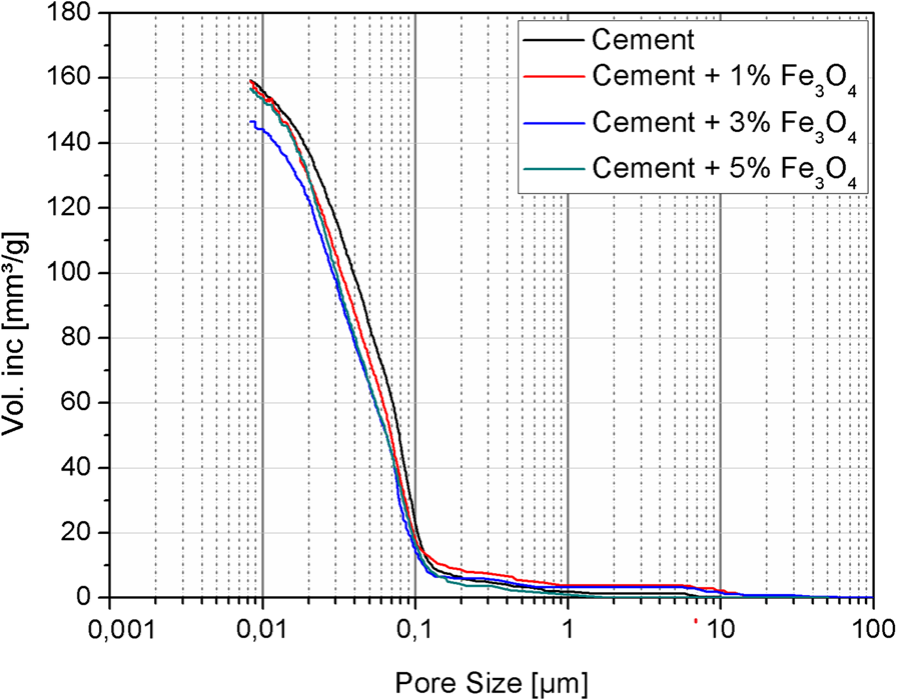
Mercury intrusion porosimetry results

The cement pastes (their matrices) were analysed also using the MIP method. The results of the MIP analysis are depicted in Fig. 6 and Table 2. The improvement of the microstructure of the cement pastes due to the presence of the nanoparticles is observed. This conclusion is supported by the reduction of the total porosity, related to the increase of the density. Moreover, a refinement of the structure can be noticed in the reduction of the average pore radius and the median pore radius size. Although an overall positive effect of the nano-Fe_3_O_4_ is present, the total porosity decreases in the following order: R > N1 > N5 > N3. This improvement can be associated with the fact that when the amount of nanofiller exceeds the optimal value, total porosity increases. However, the total porosity observed for N5 (23.89 %) is still lower than for the reference sample (25.32 %). The study has shown that 3 wt.% of nano-Fe_3_O_4_ (sample N3) is the optimal amount for the improving of the microstructure of cementitious composite (total porosity reduced to 23.78 %).

**Table 2:** Porosities, average pore radius and median radius of the R, N1, N3 and N5 samples

Sample designation	Porosity [%]	Average pore radius [nm]	Median pore radius [nm]
R	25.32 ± 1.4	18.4 ± 2.1	26.6
N1	24.98 ± 2.0	18.2 ± 2.2	26.5
N3	23.78 ± 1.4	16.5 ± 1.7	21.8
N5	23.89 ± 1.5	16.1 ± 1.8	20.6

The above described observations can also be related to strength test results, where due to microcracks caused by a high amount of nanomagnetite, a slight decrement in the flexural strength is noticed (up to 9 % for sample N5). This effect was observed especially in the flexural strength testing, because the effect of microcracks is greater on the resulting flexural strength rather than on the compressive strength [11]. In addition, a compressive strength improvement (up to 20 %) was also observed until the optimal amount of the admixture (N3), although the remaining samples (N4 and N5) were still higher than the pristine reference samples R. The presence of nano-Fe_3_O_4_ can be beneficial for the microstructure and the mechanical properties of cement-based composites. In our study, the effect of the nanoparticles during the hydration process, where only slight changes in the first days of hydration were noticed, was not very significant comparing to the available data [15]. This can be attributed to the different diameters of the tested nano-Fe_3_O_4_. In the presented study, the size of the nanoparticles was in the range of 50 to 100 nm; whereas in the current state-of-the-art, the nanomagnetic fluid contains the particles with a mean diameter of 4–7 nm, which corresponds up to 0.3 % of the mass of the cement. It is known that with a smaller particle size (and a higher surface area) more nanoparticles are available for the potential participation during the hydration process [9]. In addition, due to their magnetic properties, the nanomagnetite particles have a tendency to agglomerate [11]. Therefore, small amounts of nanomaterial with a low particle diameter can contribute to a slight improvement in the hydration process.

**Fig. 7:**
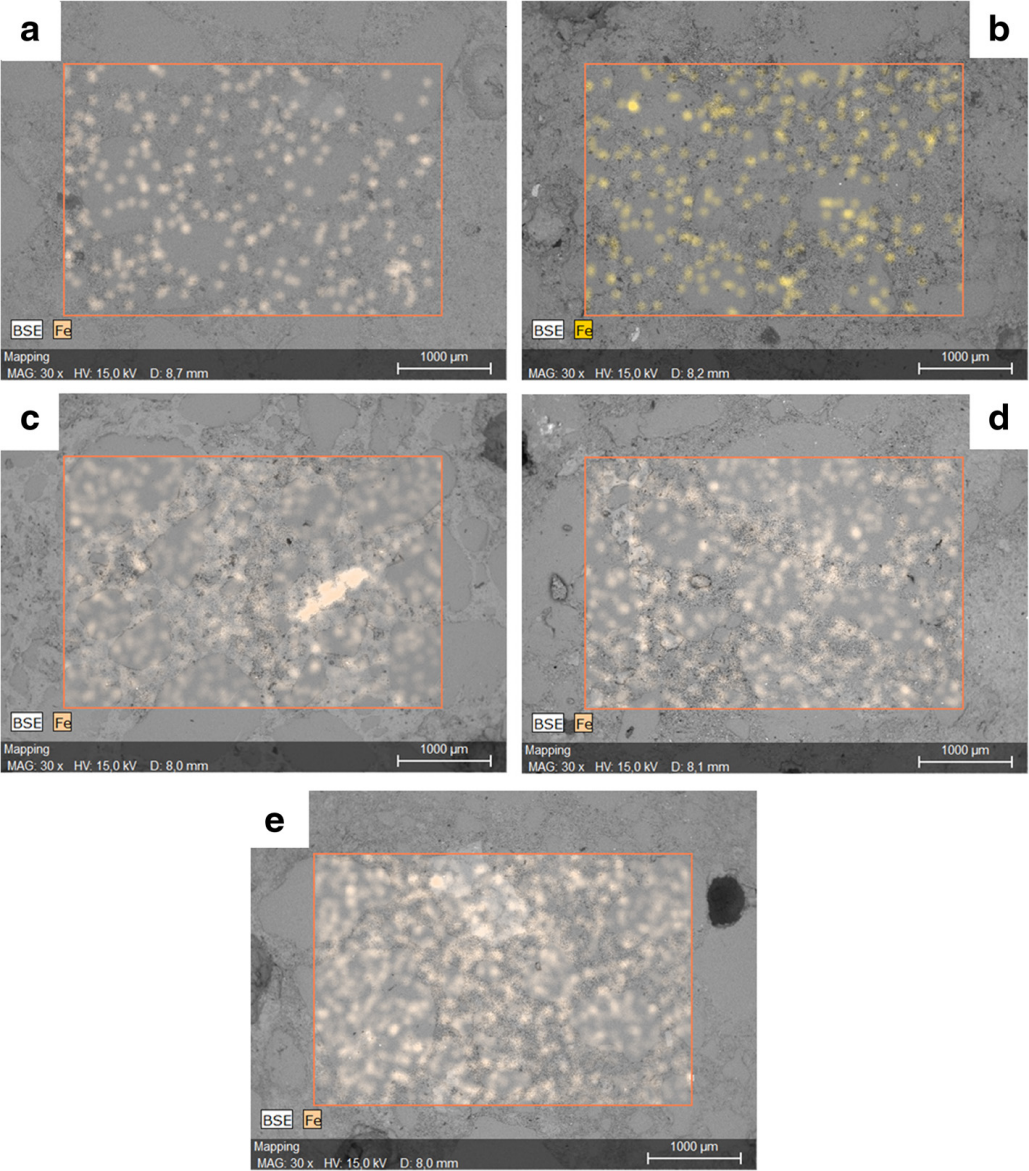
Iron distribution on the surface of the cement mortar containing different ratios of nano-Fe_3_O_4_ - 1 wt%(**a**); 2 wt%(**b**); 3 wt%(**c**); 4 wt%(**d**) and 5 wt%(**e**)

The agglomeration effect of the iron oxide nanoparticles was observed during the EDX analysis of the cement mortar. The elemental mapping and the SEM images (BSE images) are presented in Fig. 7. It can be noticed that with the increase of the nano-Fe_3_O_4_, the iron element is clearer and small agglomerations are formed. However, they are still relatively uniformly dispersed in the cement matrix. In N3, a noticeable agglomeration can be observed. Due to the magnetic properties of Fe_3_O_4_, the nanostructures clearly possess a tendency to create local agglomerations even after sonication. Therefore, due to the high amount of local agglomerations of the nanoparticles, the positive effect of the nano-Fe_3_O_4_ on the properties of the cementitious composites can be mostly attributed to the filling effect of the material, rather than to its active participation in the hydration process.

**Fig. 8:**
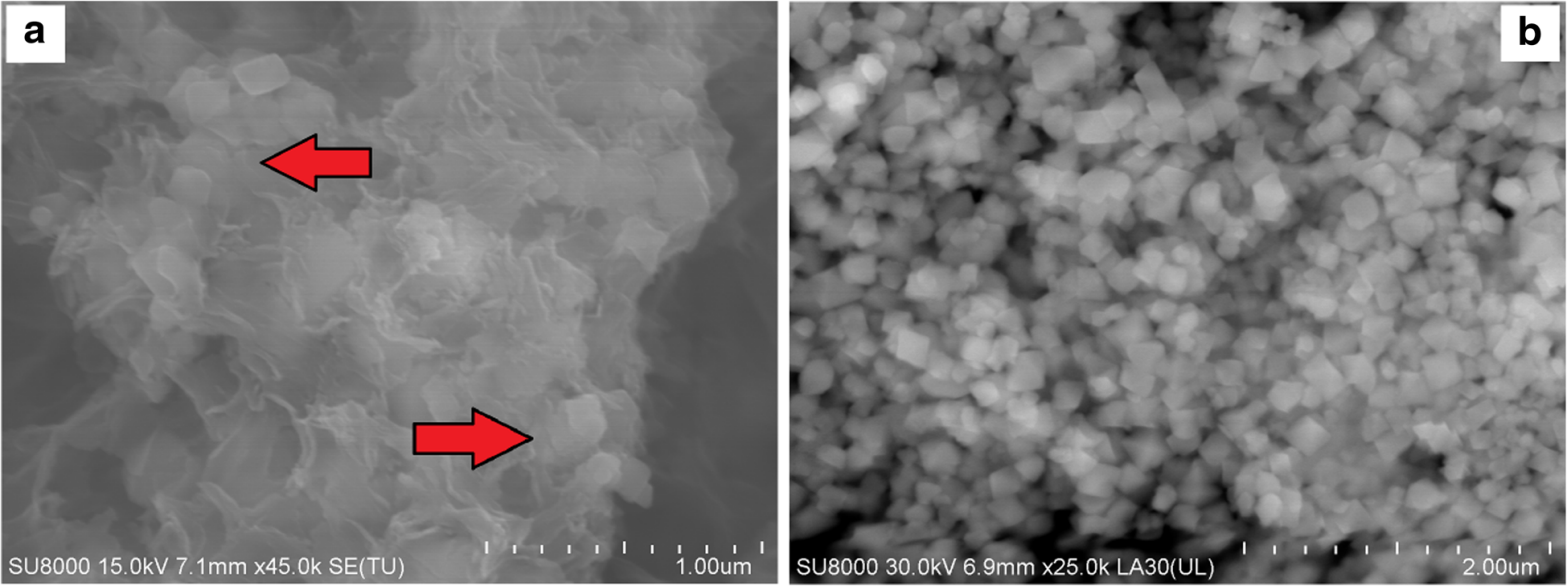
Nano-Fe_3_O_4_ in the cement composite (**a**) and reference image of the nano-Fe_3_O_4_ (**b**)

For the identification of the iron oxide nanoparticles in the cement matrix, the sample containing the optimal amount of nanomagnetite (N3) was analysed (Fig. 7c). A detailed SEM analysis of the cement paste containing nanomagnetite proves that the iron oxide nanoparticles are integrated into the cement matrix, Fig. 8a. The reference images of the iron oxide nanoparticles are presented in Fig. 8b. According to the EDX analysis, the size and shape of the individual and agglomerated nanoparticles were identified and marked with the red arrows.

## Conclusions

The experiments conducted were aimed to evaluate the effect of nanomagnetite content on the microstructure of cementitious composites. From microscopic analysis, it can be concluded that there is an optimal amount of the nanomaterials which can be successfully applied in a cementitious composite to improve its final properties. In the presence of nano-Fe_3_O_4_, a positive effect on the microstructure of the cementitious composite is noticeable; however, exceeding a certain limit might lead to the creation of local agglomerations of magnetic nanoparticles, which might not be beneficial for the mechanical response of the cement matrix. The following conclusions can be drawn:

1. nanomagnetite additive does not affect the consistency of the fresh mortars when it is applied up to 5 wt.% of the cement;
2. nanomagnetite can act as a filler of the microstructure of cement pastes by refining the pore structure and reducing the total porosity, thus increasing the density of the composite;
3. Fe_3_O_4_ nanoparticles can be successfully applied as an admixture for cementitious materials and its presence does not affect the rate of the cement hydration and the nature of the phases in hydrated cement paste;
4. nano-Fe_3_O_4_ particles have a tendency towards agglomeration; therefore, high amounts of the admixture might lead to local agglomerations and microcrack formation, what is responsible for a deterioration in the mechanical properties of cement-based composites;
5. there is a certain amount of nano-Fe_3_O_4_ (3 wt.%) which can be beneficial for the properties of cementitious composites. Exceeding this limit might lead to a neutralisation of the positive effect of the nanoparticles.
